# Innate Immunity in Diabetic Wound Healing: Focus on the Mastermind Hidden in Chronic Inflammatory

**DOI:** 10.3389/fphar.2021.653940

**Published:** 2021-04-21

**Authors:** Kang Geng, Xiumei Ma, Zongzhe Jiang, Wei Huang, Chenlin Gao, Yueli Pu, Lifang Luo, Youhua Xu, Yong Xu

**Affiliations:** ^1^ Faculty of Chinese Medicine, Macau University of Science and Technology, Avenida Wai Long, Taipa, China; ^2^ State Key Laboratory of Quality Research in Chinese Medicine (Macau University of Science and Technology), Avenida Wai Long, Taipa, China; ^3^ Cardiovascular and Metabolic Diseases Key Laboratory of Luzhou, Luzhou, China; ^4^ Sichuan Clinical Research Center for Nephropathy, Luzhou, China; ^5^ Department of Plastic and Burn Surgery, The Affiliated Hospital of Southwest Medical University, Luzhou, China; ^6^ National Key Clinical Construction Specialty, Luzhou, China; ^7^ Department of Endocrinology and Metabolism, The Affiliated Hospital of Southwest Medical University, Luzhou, China

**Keywords:** innate immunity, diabetic wound, wound healing, inflammation, senescence

## Abstract

A growing body of evidence suggests that the interaction between immune and metabolic responses is essential for maintaining tissue and organ homeostasis. These interacting disorders contribute to the development of chronic diseases associated with immune-aging such as diabetes, obesity, atherosclerosis, and nonalcoholic fatty liver disease. In Diabetic wound (DW), innate immune cells respond to the Pathogen-associated molecular patterns (PAMAs) and/or Damage-associated molecular patterns (DAMPs), changes from resting to an active phenotype, and play an important role in the triggering and maintenance of inflammation. Furthermore, the abnormal activation of innate immune pathways secondary to immune-aging also plays a key role in DW healing. Here, we review studies of innate immune cellular molecular events that identify metabolic disorders in the local microenvironment of DW and provide a historical perspective. At the same time, we describe some of the recent progress, such as TLR receptor-mediated intracellular signaling pathways that lead to the activation of NF-κB and the production of various pro-inflammatory mediators, NLRP3 inflammatory via pyroptosis, induction of IL-1β and IL-18, cGAS-STING responds to mitochondrial injury and endoplasmic reticulum stress, links sensing of metabolic stress to activation of pro-inflammatory cascades. Besides, JAK-STAT is also involved in DW healing by mediating the action of various innate immune effectors. Finally, we discuss the great potential of targeting these innate immune pathways and reprogramming innate immune cell phenotypes in DW therapy.

## Introduction

Diabetes mellitus (DM) is a chronic metabolic, endocrine disease characterized by persistent hyperglycemia. Chronic complications are its main hazards, often involving microvessels, large vessels, and the nervous system, resulting in heart, brain, kidney, and other essential organ lesions (Brennan et al., 2019; Tellechea et al., 2020). Besides, peripheral vascular and neuropathy lesions are often combined with pathogenic microbial infections to cause diabetic foot disease. According to the IDF global report in 2019, foot complications have been one of the most severe and expensive treatment complications of DM (https://diabetesatlas.org/en/sections/individual-social-and-economic-impact.html).and affect 40 to 60 million people. As the most common manifestation of foot complications, DFUs have a lifetime risk of up to 25% in DM patients (Rahelic, 2016; Ogurtsova et al., 2017; Cho et al., 2018). Continuous inflammation activation is not only the primary cause of chronic refractory DW, but also an essential factor leading to DFUs, gangrene, and amputation (toe), and even the root cause of the increased length of hospitalization and cost of wound management.

The pathogenesis of DW is complex and involves many different pathways. Although it is traditionally believed to be related to the local hyperglycemic environment, accumulation of advanced glycation end products (AGEs), oxidative stress injury, chronic inflammation, etc., more and more evidence points to the key roles of Cellular Senescence and immune aging in DW healing (Tomic-Canic and DiPietro, 2019; Berlanga-Acosta et al., 2020). Cellular Senescence is a normal physiological process in which cells lose their ability to proliferate, the essence of DW is chronic low-grade inflammation and an increased burden of senescent cells (Prattichizzo et al., 2018). In Non-DW, transient induction of a senescent phenotype such as senescent fibroblasts and endothelial cells appear very early in response to a cutaneous wound, where they accelerate wound closure by inducing myofibroblast differentiation through the secretion of platelet-derived growth factor AA (PDGF-AA) (Demaria et al., 2014), but in DW, senescent cells rapidly accumulate, these greater numbers of senescent cells and senescence-associated secretory phenotype (SASP).paly a negative role in DW healing (Wilkinson et al., 2019). Firstly, senescence is a known consequence of hyperglycemia, the direct association between hyperglycemia and SASP in endothelial cells and macrophages also suggests that SASP may exacerbate low-grade inflammation in diabetes (Prattichizzo et al., 2018). Secondly, AGEs and increased oxidative stress which lead to endoplasmic reticulum stress also promote cell senescence (Liu et al., 2014). Besides, bone marrow (BM).and mesenchymal stem cells (BMSCs).become prematurely senescent under the pressure of diabetes metabolic stress, continued release of inflammatory cytokines resulted in the loss of BMSCs number and function, which prevented DW healing (Lu et al., 2016). All of this suggests that in DW, hyperglycemia, AGEs, oxidative stress, DNA damage, inflammatory cytokines act as major drivers sculpturing the senescent phenotype (Longo et al., 2019; Palmer et al., 2019). This is reflected not only in the reduced number of cytokines and growth factors secreted and the number of receptors but also in the decline of non-functional intracellular signals (Telgenhoff and Shroot, 2005), It further affects the function of T cells and B cells, increasing susceptibility, and SASP continuously releases “inflammatory” signals to activate the innate immune system, aggravating the level of inflammatory cytokines and the secretion of cytotoxic mediators. For example, the decrease of Naive T-cell phenotype and the increase of memory and effector T-cell phenotype (Moura et al., 2017), the level of B-cell activating factor (BAFF) which implicit in B-cell dysfunction increased gradually with the progression of DFU, and the circulating BAFF was positively correlated with C-reactive protein (CRP) and TNF-α (Dhamodharan et al., 2019) (Table 1). This review focuses on the changes in innate immune cells from dormant to active phenotype in DW, to further clarify its effect on DW sustained activation of inflammation, and then discusses several kinds of immune pathways that the pattern recognition receptors or cell surface receptor trigger inflammation-related: Toll-like receptor (TLR) signal, nucleotide-binding oligomerization domain (NOD) receptor (NLR) signals, cGAS - STING signals, JAK-STAT signals, Focusing on the progress of the immune mechanisms behind chronic inflammation in DW, we finally comment on the potential of targeting these innate immune-inflammatory pathways in DW therapy. Focusing on the progress of the immune mechanisms behind chronic inflammation in DW, we finally comment on the potential of targeting these innate immune-inflammatory pathways in DW therapy.

**TABLE 1:** Markers of immune cells that can be used for diagnosis and/or treatment in DW.

Immune cells	Differentiation markers	Diagnosis and/or treatment
T Cells	Effector T-cells↑	The accumulation of effector T-cells is the core of DFU, diminish T-cells activation and tissue accumulation may accelerate DW healing Moura et al. (2017)
	Memory T-cells↑	
	Naive T-cells↓	
B Cells	BAFF↑	The BAFF levels were superior to that of CRP levels in diagnosing DFUDhamodharan et al. (2019)
Neutrophils (PMNs).	NETs↑	PAD4 inhibition and cleavage of NETs by dnaseⅠmay improve DW healing Wong et al. (2015)
	PAD4, H3Cit↑	
	NET-specific markers	NET-specific markers H3Cit negatively correlated with wound healing in DFU patients Yang et al. (2020)
	H3Cit↑	
	C5a↑	Inhibitor of complement C1 ( PIC1). may reduce the infiltration of PMNS and improve DW healing Cunnion et al. (2017)
	C3-fragment deposition↑	
Monocytes/Macrophages (Mo/Mp).	Proportions of bone MyP↑circulating Ly6C^Hi^ Mo↑	Myeloid lineage commitment in BM may contribute to increased mp numbers observed in DW strategies to regulate monopoiesis during homeostasis or post wounding may improve DW healing Barman et al. (2019)
	Spleen Ly6C^Hi^ Mo↑	
	HSPC response↑	
	MPP2, MMP-3↓	
	The influx of Ly6C^Hi^ Mo↑	Time-dependent control of Mo/Mp influx after an injury such as anti-mcp-1 antibody may represent a novel therapeutic target for impaired DW healing Kimball et al. (2018)
	Maturation to Ly6C^Lo^ Mo↓	
	IL-1β, MMP-9, TNF-α↑	Inhibiting IL-1β downregulate proinflammatory mp and upregulate prohealing mp in DW which may improve DW healing Mirza et al. (2013)
	CD206, IGF-1↓	
	TGF-β, il-10↓	
	CD68, iNOS, TNF-α, IFN-γ↑	Blocking the AGE-RAGE interaction may improve the function of Mp Wang Q. et al. (2017)
	CD206, PDGF↓	
	Jmjd3, IL-12↑	Histone demethylase inhibitor such as GSK-J4 may improve chronic inflammation and DW healing Gallagher et al. (2015)
	H3K27me3↓	
	UA, XO↑	IFNβ may be an attractive therapeutic target and XO inhibitors such as allopurinol may reduce the production of IL-1β and improve DW healing Kimball et al. (2019)
	Setdb2, H3K9me3↓	
	IFNβ↓	
	Mp senescence↑	CXCR2 antagonist treatment such as SB265610 reduces inflammation Immune-aging and improve DW healing Wilkinson et al. (2019)
	CXCR2↑	
	SASP↑	

Partial Abbreviations: BAFF: B-cell activating factor, PAD4: Peptidylarginine deiminase 4 (encoded by Padi4 in mice)., H3Cit: Citrullinated histone H3, NETs: Neutrophil extracellular traps, BM: Bone marrow, HSPC: Hematopoietic stem and progenitor cell, MyP: Marrow myeloid progenitors, MPP: Multipotent progenitor, Ly6C^Hi^: CX3CR1^low^CCR2^+^Ly6C^+^, Ly6C^Lo^: CX3CR1^high^CCR2^-^Ly6C^−^, IL-1β: Interleukin-1β, TNF-α: Tumor necrosis factor-α, MMP-9: Matrix metalloprotein-9, IL-10: Interleukin-10, TGF-β: Transforming growth factor-β, IGF-1: Insulin-like growth factor-1, AGEs: Advanced glycation end products, PDGF: Platelet derived growth factor, Jmjd3: JumanjiC (JmjC) domain-containing protein, UA: Uric acid, XO: Xanthine oxidase, SASP: Senescence-associated secretory phenotype.

## Continuous Activation of Innate Immunity Hidden Under Immune-Aging and Inflamm-Aging Hinders Diabetic wound Healing

Innate immune can resist the invasion of pathogens, clear out the endogenous signals released by damaged cells, and start the repair process, which helps maintain the steady-state of the body. However, excessive inflammation caused by immune hyperfunction can also lead to tissue damage. DW healing seems to be affected by the balance between “good” and “bad” inflammation. However, the essence is closely related to the balance of “immune response-inflammation resolution-normal healing” and “immune-aging-chronic inflammation-abnormal healing”.

In the state of normal circumstances, the immune response, coagulation cascade, and inflammatory reaction are activated after wound formation, immune cells (neutrophils, monocytes/macrophages, dendritic cells, mast cells, etc.) and repair cells (keratinocytes, epithelial cells, fibroblasts, endothelial cells, etc.) and extracellular matrix have significant changes, which in turn affect the subsequent proliferation, differentiation, migration, and remodeling (Criscitelli, 2018). The coagulation cascade forms a fibrin matrix rich in cytokines and growth factors through platelet aggregation and blood coagulation provides scaffolds for subsequent infiltrating cells (leukocytes, keratinocytes, fibroblasts, endothelial cells, etc.) (Gurtner et al., 2008; Minutti et al., 2017; Bando et al., 2018; Criscitelli, 2018). Then, immune cells migrate to the wound under the influence of chemokines and release a large number of cytokines to initiate the inflammatory response (Bando et al., 2018; Akita, 2019), neutrophils are first recruited to the wound and reach a peak within 24 h (Kim et al., 2008), by changing the phenotype and expression of macrophages to generate the innate immune response (Park and Barbul, 2004; Acosta et al., 2008; Suga et al., 2014). Subsequently, monocytes are recruited in the wound within 48–96 h after injury and differentiate into macrophages, carry out the function of engulfing pathogens and cellular debris, promote the secretion of cytokines, growth factors, and chemokines, stimulate collagen synthesis and angiogenesis, promote the conversion of fibroblasts to myofibroblasts to fill and contract the wound, and provide supports for subsequent cell proliferation, migration, and re-epithelialization (Snyder et al., 2016; Kim and Nair, 2019). When the granulation tissue formed by fibroblasts, endothelial cells, and macrophages replaces the fibrin matrix, keratinocytes and epithelial cells migrate on the new scaffolds until the skin barrier function is restored (Gurtner et al., 2008; Bainbridge, 2013).

Immune senescence in DW is further evidenced by reduced recruitment of immune cells and poor control of inflammatory response due to dysregulation of transcriptional networks. The study found that: Transcription factors FOXM1 and STAT3, which activate and promote the survival of immune cells, are inhibited in DFU (Sawaya et al., 2020), In the state of DM, the insufficient inflammatory response in the early stage of the wound and the large number of immune cells infiltrating in local subsequently (Moura et al., 2017), releasing proinflammatory cytokines make the healing stagnate in the inflammatory period and difficult to enter the proliferation and remodeling period (Liu et al., 2019) are the pathological basis of chronic refractory DW (Zhao et al., 2016). Moreover, innate immune cells such as neutrophils and macrophages continuously infiltrate during the inflammatory phase and interact with the high expression of chemokines, Macrophage inflammatory protein-2 (MIP-2) and Monocyte chemotactic protein-1 (MCP-1), up-regulate inflammatory mediators IL-1β, TNF-α, etc. result in continual amplification of signals of inflammatory until 13 days or more after injury (Wetzler et al., 2000; Tousoulis et al., 2013; Mo et al., 2019). Meanwhile, the continual differentiation and formation of proinflammatory macrophages can also stimulate the synthesis of Matrix metalloproteinases (MMPs) jointly with TNF-α, cause excessive destruction of extracellular Matrix and damaged granulation tissue formation (Acosta et al., 2008), inhibit proliferation and migration of fibroblasts and angiogenesis (Xu et al., 2013), activate innate immunity through ROS generated by oxidative stress (Warnatsch et al., 2015), activate NLRP3 inflammasome and lead to exacerbating wound inflammation (Lee et al., 2013; Mirza et al., 2014; Dai et al., 2017). It can be seen that the intensified innate immune response and the continuous infiltration of innate immune cells will destroy the balance between anti-inflammatory and proinflammatory during DW healing, interfere with the homeostasis of DW, and form a chronic refractory wound characterized by DFUs (Chen et al., 2012). (Table 1).

**FIGURE 1:**
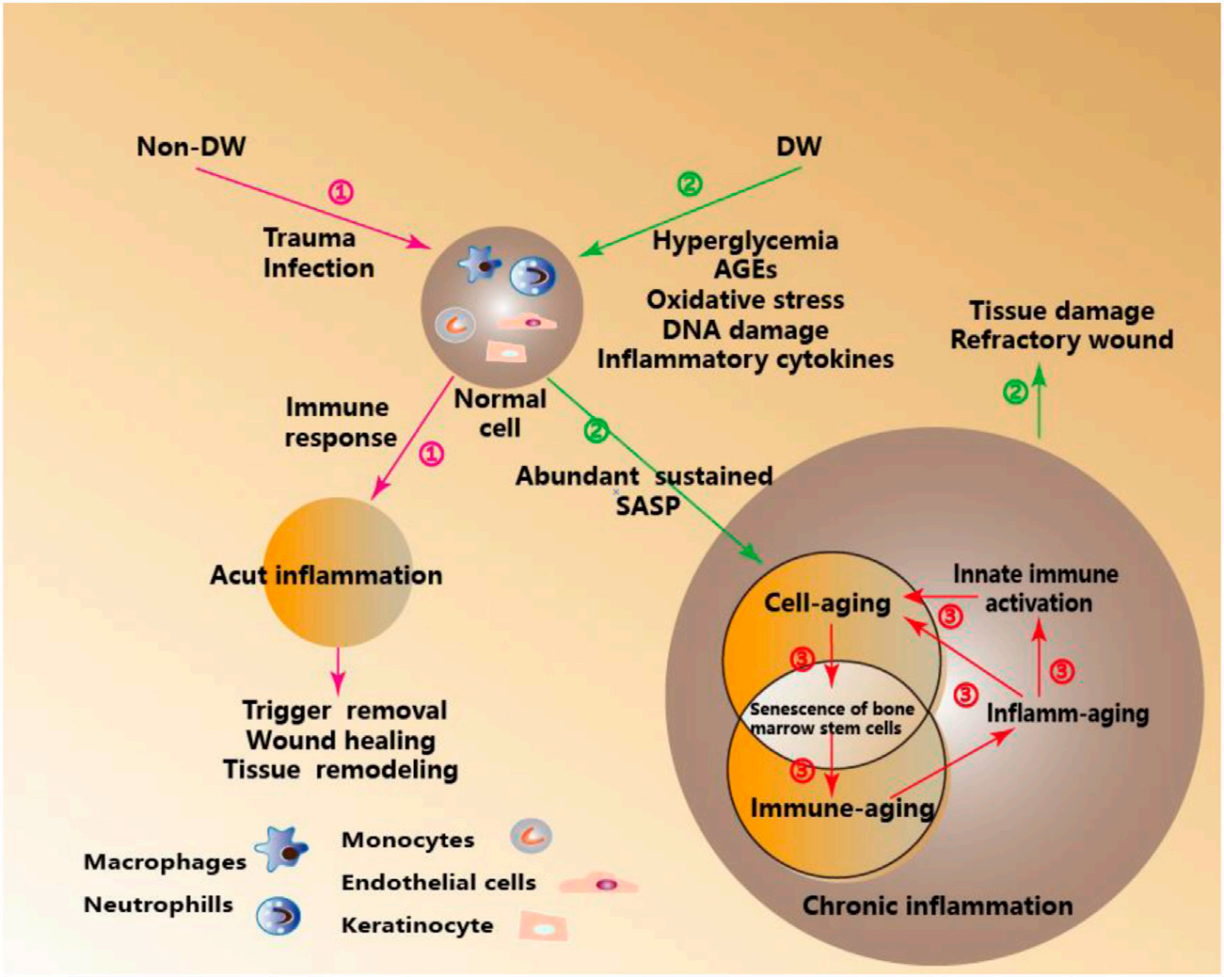
Immune- Aging and Inflamm-Aging under Metabolic Stress in DW. ①Acute inflammation-mediated wound healing, tissue remodeling, and other orderly outcomes under the immune response in Non-DW. ②Inflamm-aging induced by metabolic pressure mediated tissue damage and hinders wound healing in DW. ③In the process cell senescence in DW, adaptive immunity significantly decreased, which is called immune-aging, while innate immunity is activated, thus inducing a unique pro-inflammatory response, which is called inflamm-aging.

In a word, Inflamm-aging and Immune-aging seem to run in parallel and form a vicious cycle. Increased inflammatory cytokines characteristic of inflamm-aging contributes to the decrease of the adaptive immune response and eventually to immune-aging. In contrast, the decrease of the adaptive immune response reinforces the stimulation of the innate immune response (as the means to protect an organism from infections in the circumstances when adaptive immunity fails)., leading to chronic inflammation (Salminen et al., 2008; Fulop et al., 2017). (Figure 1).

## Innate Immune Cells Affected by Immune-Aging in Diabetic wound

### The Ongoing Recruitment and Activation of Neutrophils (Polymorphonuclear leukocytes, PMNs). and the Feedback Loop of Oxidative Stress Aggravate the Damage in Diabetic wound

PMNs phagocytize, neutralize, and remove microorganisms, cell debris, and other substances through phagocytosis, degranulation, generation of high concentrations of ROS, and Neutrophil extracellular traps (NETs) (Fox et al., 2010; Mikhalchik et al., 2018; Papayannopoulos, 2018). It can also regulate the phenotype of macrophages and the expression of cytokines to mediate the innate immune response, secrete a variety of inflammatory mediators, and an enzyme to regulate the activity of inflammatory cells and the inflammatory response (Daley et al., 2005). In the state of DM, the chemotaxis, phagocytosis, oxidative burst, and apoptosis of PMNs in DW induced by high glucose are deficient (Nguyen et al., 2013). PMNs infiltrate local tissues, combine with AGEs and release a large number of proinflammatory factors to cause inflammation. Meanwhile, PMNs promote excessive production of ROS and upregulate the expression of EGFR, ERK, and IL-8, further aggravate the degree of local infiltration (Lan et al., 2013). This continuous inflammation feedback loop driven by PMNs is an important reason for the aggravation of DW injury. Besides, the orderly resolution of PMNs is also the key to wound healing as scheduled. In the state of normal circumstances, PMNs are specially recognized and cleared by macrophages through apoptosis, to avoid entering a state of chronic inflammation and further tissue damage (Nepal et al., 2019; Perez et al., 2019). Surviving PMNs can also be “reverse-migrated” into the circulation and leave the site of inflammation by Specialized pro-resolving mediators (SPMs) (Robertson et al., 2014). However, in the state of DW, PMNs are more likely to die in the form of NETosis, forming NETs to induce innate immune cells to infiltrate the wound and mediate abnormal inflammation. Recent studies have shown that NETs can activate NLRP3 inflammasome, thus inducing the expression and release of inflammatory factors such as IL-1β and leading to inflammatory storms. After giving PAD4 inhibitor (Cl-amidine) (Fadini et al., 2016), dnaseⅠ, N-acetylcysteine (NAC) (Liu et al., 2019), targeted inhibition of the formation of NETs can inhibit the activation of NLRP3 inflammasome and the release of inflammatory mediators, reduce local infiltration of PMNs, promote macrophages appearance in advance and accelerate the resolving of PMNs, thereby promoting wound healing (Wong et al., 2015; Liu et al., 2019; Yang et al., 2020). Besides, the activation of the complement system (CS) in the early stage of DW also promotes leukocytes (the vast majority of the leukocytes were PMNS) infiltration, It was found that increased anaphylatoxin C5A and C3-fragment deposition in DW fluid associated with a 76% increase in PMNS, use the novel classical CS inhibitor, Peptide Inhibitor of Complement C1(PIC1) reduced inflammation as reflected by reduced CS components and PMNs infiltration (Kimball et al., 2018). Thus, inducing the apoptosis of PMNs, promoting the degradation of NETs, negative regulation of the complement system, and promoting the “reverse-migrated” of PMNs through SPMs will change the chaotic state of “Activation in the early period and resolving in the late period delayed” PMNs, thereby promoting DW healing.

### Spatial and Temporal Expression Difference of Monocytes Is a Double-Edged Sword for Diabetic wound Healing

Mo in peripheral blood of mice is divided into two subgroups: CX3CR1^low^ CCR2^+^ Ly6C^+^ (Ly6C^+^) and CX3CR1^high^ CCR2^−^Ly6C^−^ (Ly6C^−^). After the wound is formed, Ly6C^+^ first migrates to the wound and differentiate into macrophages and secrete pro-inflammatory factors such as TNF-α, IL-1β to participate in the early inflammatory response (Daley et al., 2010; Brancato and Albina, 2011). Subsequently, Ly6C^−^ appears on the wound through vascular patrol and is involved in anti-inflammatory and tissue repair (Carlin et al., 2013; Italiani and Boraschi, 2014). Besides, Ly6C^+^ can also induce apoptosis of PMNs, while Ly6C^−^ has the effect of removing PMNs fragments after apoptosis (Peng et al., 2009). The chemotactic activity of Ly6C^+^ is mainly dependent on CCL2/CCR2. Current studies have shown that delayed response and impaired activity of macrophages, which differentiated from Ly6C^+^ at the early stage of DW are related to decreased expression of CCL2 chemokines (Wood et al., 2014). Animal experiments have shown that the use of CCL2 on the wound of db/db mice can not only solve the delayed early inflammatory response and macrophage dysfunction, promote re-epithelialization, but also restore the amount of Mo and promote the chemotaxis of Mo (Weinheimer-Haus et al., 2014; Wood et al., 2014). Besides, knocking out the CCR2 gene can cause a decreased amount of Ly6C^+^ recruited to the wound tissue, leading to tissue repair disorders (Weinheimer-Haus et al., 2014). However, excessive expression of Ly6C + on the wound can also affect wound healing, which requires strict control of the timing of the use of CCR2. The chronic refractory DW is related to increased secretion of Ly6C+ and decreased secretion of Ly6C^−^ in circulating blood (Galstyan et al., 2019). Studies have shown that the influx of secondary mononuclear/macrophage will increase in wounds around 96 h after injury. Therefore, selective inhibition of CCL2/CCR2 at 72 h after dBinjury can improve the persistent state of DW inflammation and promote healing by blocking the second influx of Ly6C^+^ (Kimball et al., 2018). In summary, using chemokines as the target to regulate the timing of Mo appears on the wound will be expected to become an effective means to promote DW healing.

### The Persistent Proinflammatory Phenotype of Macrophages in the Metabolic Immune Microenvironment Aggravates the Difficulty in Diabetic wound Healing

Mp involved in wound healing includes Tissue-resident macrophages (TRMs). (Ganesh and Ramkumar, 2020) and Wound-associated macrophages (WAMs). (Wang et al., 2014). Skin TRMs include Langerhans cell (LC) in the *epidermis* and macrophage population (CD11b^+^/ F4/80^+^) in the dermis, through self-amplification (Chorro et al., 2009; Hoeffel et al., 2012; Sieweke and Allen, 2013) and the differentiation of circulating blood Mo in the inflammatory state (Sieweke and Allen, 2013) to supplement, play the role as a sentinel for tissue homeostasis, initiate an inflammatory response and form a locally pro-inflammatory environment by way of PRRs identifying PAMPs or DAMPs in the immediate or early stages of trauma (Smyth and Bertram, 2019). Under steady-state conditions, TRMs maintain homeostasis. Once damaged, WAMs are recruited to wound in large quantities and promote the progress of wound healing together with TRMs. With the assistance of some new techniques such as cell tracking and single-cell RNA-seq, the heterogeneity of TRMs and WAMs appears more in the study of wound healing. TRMs are considered to be involved in the induction of inflammation (Stojadinovic et al., 2013). At the same time, WAMs are not only crucial in the initial inflammation stage of wound healing but also play a vital role in the subsequent stage of wound healing by coordinating the inflammatory response (Willenborg et al., 2012). (Table 2).

**TABLE 2:** Function and relationship of Mo/Mp subpopulations in wound homeostasis and repair.

Mo/Mp	Function		
	Feature the steady-state		Wound healing
Ly6C^+^ (Sieweke and Allen, 2013)	CCL2 can regulate its chemotactic activity Willenborg et al. (2012), Gupta et al. (2013), Dal-Secco et al. (2015)	1.A precursor of the TRMs Italiani and Boraschi, (2014)	1.Upregulate TNF-α, IL-1β
			2.Activate a function similar to M1 Daley et al. (2010), Brancato and Albina, (2011)
		2.A precursor of Ly6C^−^ in blood and bone marrow Varol et al. (2007), Yona et al. (2013)	3.Die in the wound during the inflammatory, repair, proliferation period Albina et al. (1990)
			4.Enter the non-lymphoid organs and circulated to the lymph nodes Jakubzick et al. (2013)
Ly6C^−^ (Sieweke and Allen, 2013)	Fractalkine (CX3CL1) can regulate its chemotactic activity Ishida et al. (2008), Lauvau et al. (2015)	1.Patrol the signs of endothelial inflammation or injury Auffray et al. (2007), Carlin et al. (2013)	1.Upregulate TGF-β, VEGF
		2.Produce inflammatory mediators and coordinate the repair of damaged vascular endothelium Carlin et al. (2013), Italiani and Boraschi, (2014)	2.Activate a function similar to M2 Daley et al. (2010), Brancato and Albina, (2011)
TRMs F4/80^+^ (Ganesh and Ramkumar, 2020; Sieweke and Allen, 2013)	Bone marrow/Circulation Sieweke and Allen, (2013) and embryonic progenitor cells (yolk sac, fetal liver) derived Mp Chorro et al. (2009), Hoeffel et al. (2012), Sieweke and Allen, (2013)	Local proliferation and self-renewal of mature differentiated cells without changing their differentiation phenotype Chorro et al. (2009), Hashimoto et al. (2013), Sieweke and Allen, (2013)	1.Involve in the induction of inflammation Stojadinovic et al. (2013)
			2. Compensatory regulation through early recruitment and late self-proliferation Davies et al. (2011), Sieweke and Allen, (2013)
			3. Activate a function similar to M2 Brancato and Albina, (2011)
WAMs F4/80^−^ (Wang et al., 2014; Sieweke and Allen, 2013)	M1 Koh et al. (2013)	GM-CSF, IFN-γ, TNF-α, LPS induce Koh et al. (2013)	1.M1 has the function of promoting inflammation
	M2 Koh et al. (2013)	M2a: IL-4, IL-13 induce Lech et al. (2012)	2.M2 has the function of anti-inflammatory, repairing tissue, promoting angiogenesis
		M2b: Immune complexes, TLR receptor agonists, IL-1 receptor agonists induce Mantovani et al. (2004)	3.M2a promote matrix reconstruction and tissue repair
		M2c: IL-10, TGF-β, glucocorticoid induce Lech et al. (2012)	4.M2b and M2c mainly play the function of immune regulation Brancato and Albina, (2011)

Partial Abbreviations: CCL2: Chemokine C-C-motif ligand 2, TRMs: Tissue-resident macrophages, TNF-α: Tumor necrosis factor-α, IL: Interleukin, CX3CL1: Chemokine C-X3-C-motif Ligand 1, TLR: Toll-like receptor, TGF-β: Transforming growth factor-β, VEGF: Vascular endothelial growth factor, WAMs: Wound-associated macrophages, GM-CSF: Granulocyte-macrophage colony-stimulating factor, LPS: Lipopolysaccharide.

Under the influence of different microenvironments, Mp is polarized into pro-inflammatory M1 (Classically activated macrophages type 1) and anti-inflammatory M2 (Alternatively activated macrophages), which respectively play roles in different stages of wound healing (Das et al., 2015; Krzyszczyk and et al., 2018). In the stage of an inflammatory response, the wound is dominated by M1, which play an antibacterial and clearance of necrotic tissue role through phagocytosis, producing ROS, secreting various proteases and pro-inflammatory mediators (such as IL-1β, TNF-α, NO and IL-6). Thereafter, M1 gradually polarize to M2 which promote cell proliferation, Extracellular matrix (ECM) synthesis, angiogenesis, and tissue remodeling through secreting anti-inflammatory factors (such as IL-10, IL-8, etc.), various ECM proteins and growth factors (such as TGF-β1, bFGF, PDGF and VEGF, etc.) (Murray et al., 2014; Wang et al., 2014; Boniakowski et al., 2017). The orderly transformation of M1 to M2 is the key to wound healing (Ganesh and Ramkumar, 2020), If Mp cannot acquire the M2 phenotype, sustained pro-inflammatory signals will amplify the pro-inflammatory effect of M1 in the form of positive feedback by continuously activating NLRP3 inflammasome and promoting IL-1β release (Warnatsch et al., 2015). Besides, the persistent state of inflammation caused by such phenotypic imbalance of Mp also hinders the proliferation and migration of endothelial cells and keratinocytes, prevents fibroblasts from secreting extracellular matrix, and secretes a large number of proteases to degrade the extracellular matrix (Pierce, 2001), these degraded extracellular matrix fragments further aggravate the pro-inflammatory state of the wound through the action of immune stimulation (Sorokin, 2010). Clinical studies have shown that Mp isolated from DW express high levels of pro-inflammatory molecules IL-1β, MMP-9 and TNF-α and low levels of healing-related molecules IGF-1, TGF-β, and IL-10. A similar phenomenon is also observed in animal experiments. Mp in Non-DM mice shows M2 phenotype 5–10°days after injury while DM mice maintain the M1 phenotype. Also, the IL-1β and TNF-α released by DM mice Mp on the fifth and 10th°day after injury are always maintained at high levels, and IGF-1, TGF-β are maintained at low levels. IL-1β neutralizing antibody or knocking out IL-1R1 gene can block IL-1β signal, thereby down-regulating M1, up-regulating M2, and promoting DW healing (Mirza et al., 2013).

The act of PMNs clearance by Mp can induce the phenotypic switch of M1 macrophages to M2, hyperglycemia, and AGEs impede the phagocytic capacity of Mp to clear apoptotic PMNs thereby promoting a sustained pro-inflammatory state (Hesketh et al., 2017). Further, differential iron regulation by Mp is another factor that affects Mp phenotype (Cairo et al., 2011; Wang H. et al., 2017), iron overloading in Mp maintaining M1 phenotype, enhanced TNF-α and hydroxyl radical release and induce nearby fibroblasts senescence. In addition to the cellular and molecular mechanisms of the effect of microenvironment on the Mp phenotype, the effect of epigenetics has also become a new focus of recent research, Skin wounding increased HSPC numbers and promoted Mo expansion in the BM of mice (Barman et al., 2019), even hyperglycemia preprogrammed HSPCs toward myeloid lineage commitment (Nagareddy et al., 2013), diabetic mice exhibited increased proportions of bone MyP and circulating inflammatory Mo before skin wounding and enhanced myeloid output maintained following injury, contributing to a greater number of M1 phenotype (Barman et al., 2019). Further, Histone methylation can also alter the Mp phenotype to affect DW healing (Boniakowski et al., 2017). For example, MLL1-mediated epigenetic alterations activated H3K4me3-TLR4-MyD88, use TLR4 inhibitor TAK-242 as well as genetic depletion of either TLR4^−/−^ or myeloid-specific TLR4^f/f^Lyz2^Cre+^ resulted in a reduction in Mp-mediated inflammation and improved DW healing (Davis et al., 2020). In another study, the decreased expression of H3K27me3 corresponds to the increased Jmjd3 and IL12 expression promoting the exaggerated pro-inflammatory response seen in diabetic Mp (Gallagher et al., 2015). This suggested that both target systemic changes in the BM stem cells thus influence Mp peripheral phenotypes and blocking the M1 phenotype in DW may contribute to the development of new therapeutic strategies for DW and is a promising research direction.

### The Homeostasis of Mast cell Degranulation Regulates Ordered Healing in Diabetic wound

After wound formation, MCs participate in and regulate the inflammatory response by releasing multiple mediators (Moon et al., 2010). On the one hand, MCs induce the secretion of cytokines and chemokines by degranulation (Sun et al., 2012; Mukai et al., 2018; Zelechowska et al., 2018), On the other hand, MCs recruit immune cells to the wound by promoting the secretion of vascular permeability factors and proteases (Liu et al., 2009). Subsequently, MCs can stimulate the proliferation of fibroblasts by secreting IL-4, VEGF, and bFGF (Qu et al., 1998), promote granulation formation, cell migration, angiogenesis, collagen maturation, and angiogenesis in wound tissues (Sismanopoulos et al., 2012; González-de-Olano and Álvarez-Twose, 2018), and also participate in wound healing with Mp, endothelial cells and fibroblasts (Egozi et al., 2003; Nishida et al., 2019). Current clinical studies have shown that the total MCs before lower limb skin trauma in DM patients are regular, but the number of degranulation is increasing, and MCs degranulation is positively correlated with the number of dermal inflammatory cells and inflammatory markers IL-6 and TNF-α (Tellechea et al., 2016). Animal experiments have shown that intraperitoneal injection of DSCG (MCs degranulation inhibitor) before trauma can reduce the number of MCs degranulation in DM mice to the same level as non-DM mice, and effectively improve wound healing. Besides, DSCG can restore the ratio of M1/M2 to the level of non-DW through the interaction between MCs and Mp, and promote wound healing (Tellechea et al., 2016). In addition to the difference in pre-trauma degranulation, the current studies also have found that: in the state of diabetes, although MCs degranulation increased significantly before the trauma, it cannot increase after trauma, and the blocked MCs degranulation also reduces the ability of acute inflammatory response after trauma and delays the process of wound healing. In the early stage of wound formation, the local application of MCs stabilizer MCS-01 to treat DM mice can play the same role as the intraperitoneal injection of DSCG to promote wound healing (Tellechea et al., 2020). It can be seen that maintaining the steady-state of degranulation of MCs before trauma and degranulation in time after trauma will effectively promote DW healing.

## Innate Immune Signaling and Diabetic wound

**FIGURE 2:**
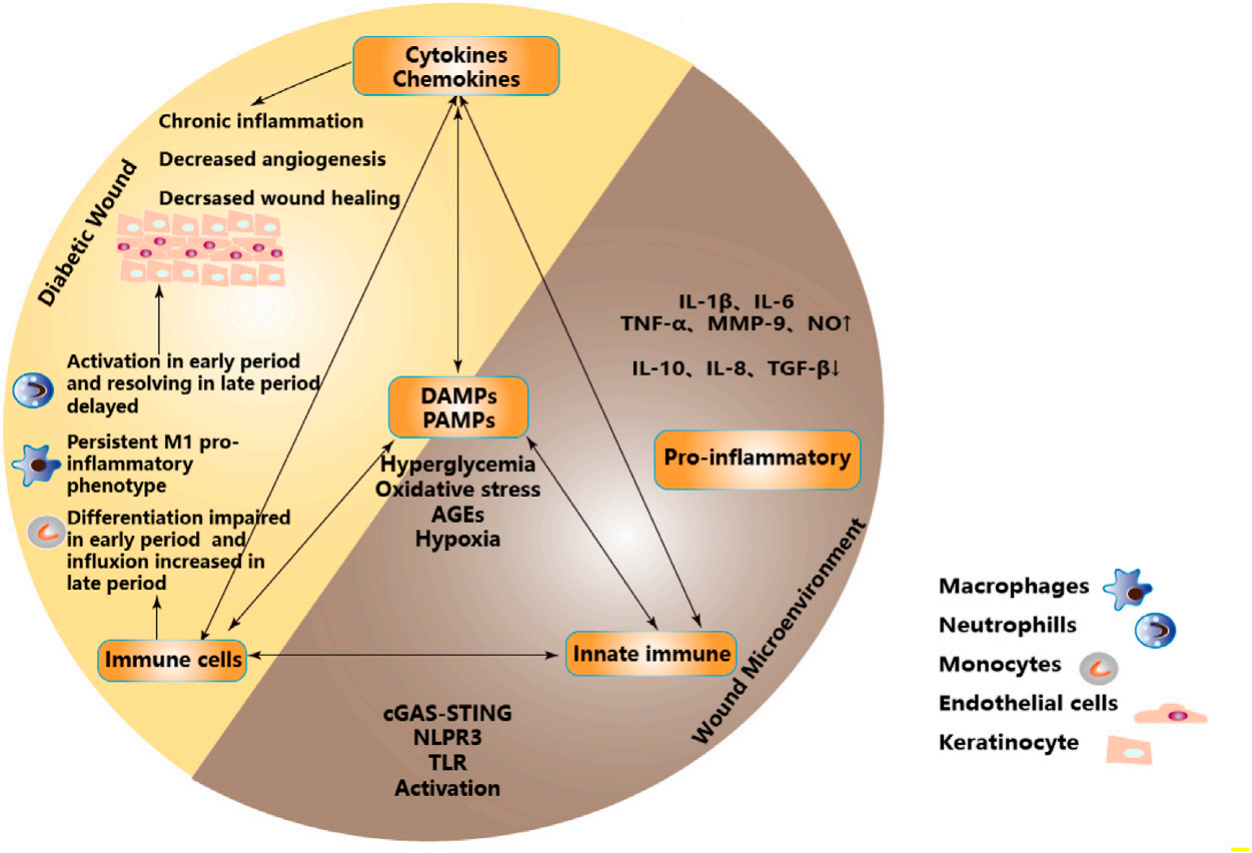
Proinflammatory microenvironment in DW due to dysregulation of immune and metabolic responses. Metabolic stress such as hyperglycemia, oxidative stress, AGEs, and hypoxia acted as the initiators of PAMPs and DAMPs to activate PRRs. Inducing infiltration of innate immune cells and activation of innate immune pathways, regulating the balance between innate immunity and inflammatory microenvironment, forming a pro-inflammatory microenvironment, and participating in DW healing. Partial abbreviation: IL-1β: Interleukin-1β, IL-6: Interleukin-6, TNF-α: Tumor necrosis factor-α, MMP-9: Matrix metalloprotein-9, NO: Nitric oxide, IL-10: Interleukin-10, IL-8: Interleukin-8, TGF-β: Transforming growth factor-β, DAMPs: Damage-associated molecular patterns, PAMPs: Pathogen-associated molecular patterns, TLR: Toll-like receptor.

In the state of DW, the “abnormal epithelial barrier” caused by changes of microbiome located in the skin and their metabolites, environmental damage or the genetic tendency of the host, as well as the cellular contents released after necrosis and cell membrane destruction caused by stress, injury and metabolic pressure, recruit and activate innate immune cells and initiate innate immunity not only through intracellular DAMPs but also through extracellular DAMPs and PAMPs released by extracellular matrix recruitment (Matzinger, 2002; Huebener and Schwabe, 2013). When the activated innate immunity plays a role in removing pathogens and necrotic tissues, the high concentration of ROS produced also cause lipid peroxidation damage to the cell membrane, increase membrane permeability, destroy the critical balance of ion concentration inside and outside the cell, and further aggravate the release of DAMPs (Mayer et al., 2018). This subtle relationship as initiating factors regulate the balance between innate immunity and pro-inflammatory microenvironment and participate in the process of DW healing. It is necessary to understand further these PRRs that recognize PAMPs or DAMPs, including transmembrane receptors on the cell membrane surface, such as Toll-like receptor(TLR), C-type lectin receptor(CLR), etc. and intracytoplasmic receptor, such as RIG-1-like receptor(RLR), Nucleotide-binding oligomerization domain-like receptor (NLR).etc. And Cytosolic DNA Sensor (CDS) , such as cGAS, STING, etc. (Figure 2)

### Increased Toll-Like Receptor Expression and Activation Prolonged Inflammatory Condition in Diabetic wound

**FIGURE 3:**
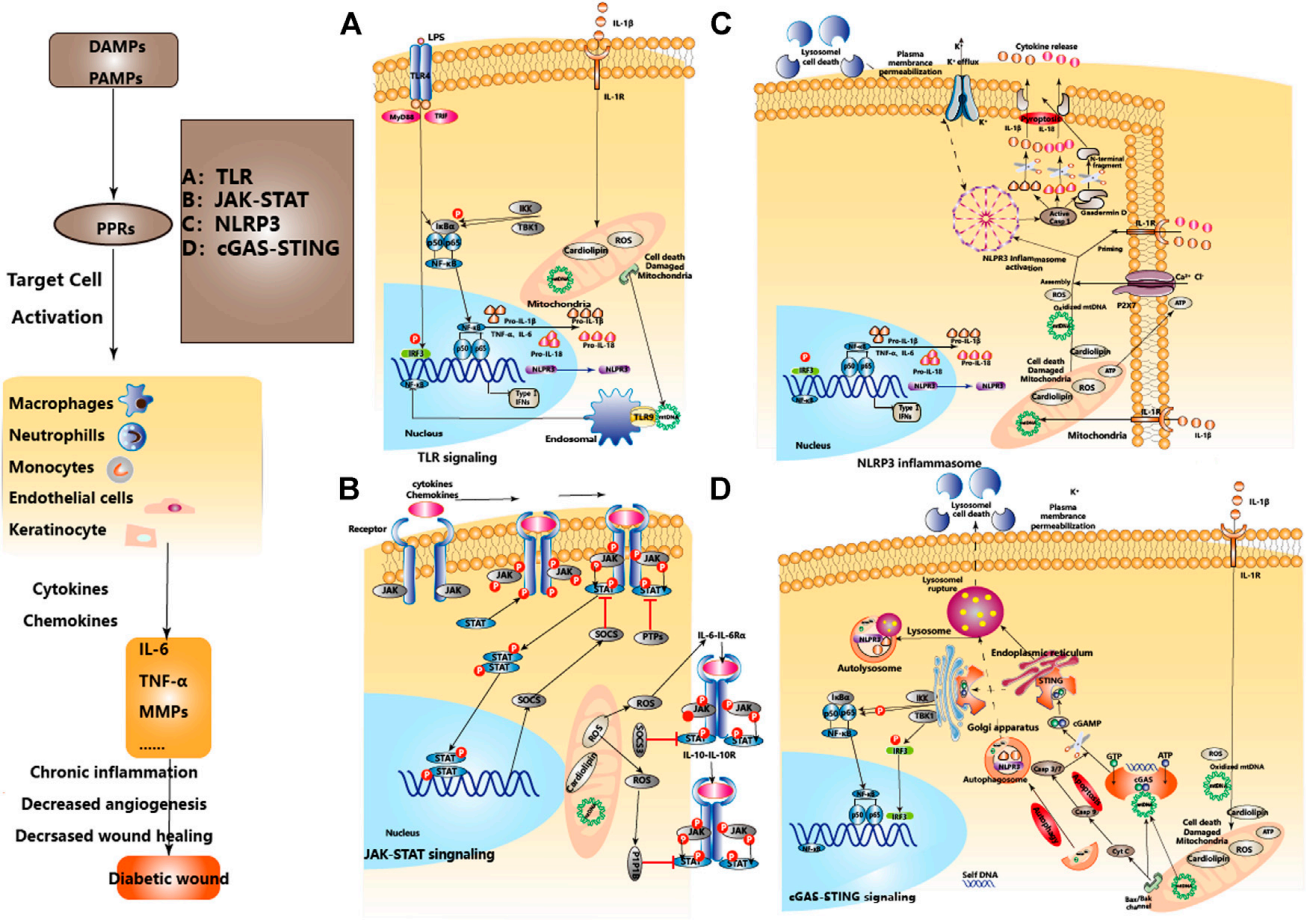
The regulatory relationship between different innate immune pathways in DW and homeostasis. The effects of the regulatory relationship between TLR, JAK-STAT, NLPR3, cGAS-STING signal pathways, and homeostasis in DW. After the formation of DW, PAMPs and DAMPs activate PRRs as initiating factors, inducing the activation of TLR, NLPR3, and cGAS-STING signal pathways, activate the expression of type I interferon and other immunoregulatory molecules. JAK-STAT mediates extracellular-nuclear regulation of various cytokines/chemokines. Mitochondrial damage, endoplasmic reticulum stress, and lysosomal membrane permeabilization are involved in the activation and regulation of signal pathways. Mitochondrial autophagy and apoptosis play an immune silencing role in maintaining immune homeostasis. A, B, C, and D represent four signaling pathways respectively.

TLR can respond to various pathogens (such as bacterial LPS, viral double-stranded RNA, etc.) or endogenous signals released after cellular stress and injury (such as HSP, HMGB1, Hyaluronic acid, Fibrinogen, etc.), and play a key role in inflammation, immune cell regulation and proliferation (Huebener and Schwabe, 2013). TLR signaling can be divided into MyD88-dependent pathway and TRIF-dependent pathway, the former mainly activates NF-κB and MAPKs, while the latter mainly activates NF-κB and IRF3(Kawai and Akira, 2007). The activation time of TLR binding with ligands and its relationship with the local microenvironment determine the effect of TLR in DW (Dasu and Rivkah Isseroff, 2012; Portou et al., 2015). In Non-DW, the expressions of TLR2 and TLR4 were up-regulated in the inflammatory stage (Suga et al., 2014), gradually decreased after the repair stage and recovered to the baseline level in 10°days later (Chen et al., 2013), regulates the release of IL-1β and IL-6, and participates in the orderly wound healing via TLR4-p38/JNK-MAPK signaling. But in DW, TLR2, 4, and 6 were consistently highly expressed from the injury to the 10th°day, continuously activated the NF-κB signal axis, and continuously amplified the pro-inflammatory signals such as IL-1β, IL-6, and TNF-α, which hindered the healing of DW (Dasu et al., 2010; Singh et al., 2015). More studies have also pointed to the negative regulatory role of abnormal inflammation caused by continued activation of TLR3, 7, and 9 in DW healing (Singh et al., 2016; Wu et al., 2016). Clinical studies of DW have also found that the levels of TLR1, 2, 4, and 6 and corresponding expression of downstream adapter proteins such as MyD88, IRAK-1 and NF-κB in DW were significantly higher than those in Non-DW (Dasu and Martin, 2014). This shows activated TLR-MyD88-NF-κB signaling and increased oxidative stress contributes to the increased local pro-inflammatory cytokines and unhealed DW. Besides, high glucose up-regulated transcription and translation of TLR2 and TLR4 in a time-dependent and dose-dependent manner, inducing heterodimerization of TLR2/TLR6, leading to recruitment of MyD88 and activation of downstream inflammatory pathways, and the secretion of IL-1β and TNF-α(Dasu et al., 2008)keeping DW in an inflammatory state (Dasu and Martin, 2014; Kishibe et al., 2018), while inhibiting TLR2 and TLR4 can block the excessive inflammation caused by the positive feedback effect of IL-1β, thereby promoting DW healing (Dasu et al., 2010). (Figure 3A).

### Therapeutic Targeting of Toll-Like Receptor Signaling in Diabetic wound

In keratinocytes, hyperglycemia dose-dependently up-regulates the expression of TNF-α to reduce the expression of Thrombomodulin (TM) and TLR4, exogenous supplementation of Recombinant sTM can increase the expression of TLR4 and promote DW healing (Cheng et al., 2015). Besides, hyperglycemia can also inhibit the expression of IL-33, which is induced by IL-17 in keratinocytes through glycosylation, down-regulate Regenerating islet-derived protein 3A (REG3A), and up-regulate the expression of TLR3, supplementing REG3A can induce SHP-1 negatively regulating the TLR3-JNK2 signal axis to down-regulate TNF-α and IL-6, thereby improving chronic inflammation of DW (Wu et al., 2016). However, the study of Mo has found that using NADPH and PKC inhibitors to down-regulate the expression of TLR2 and TLR4 can improve the continuous inflammation induced by hyperglycemia (Dasu et al., 2008). It is suggested that targeting TLR2 and 4 can reduce the release of inflammatory signals IL-1β and TNF-α, improve the sustained inflammatory state of the wound surface, and also show the effective role of targeting TLR signaling in different effector cells of DW. Besides, PPARγ agonists can also down-regulate the expression of TLR4 (Ji et al., 2009). Using Pioglitazone-loaded fibrous mats which are prepared from pioglitazone hydrochloride (PHR) on the wound of DM rats, it is found that both burst-release and sustained-release kinetics can down-regulate the expression of TNF-α and promote DW healing, but the sustained-release form is more effective in reducing PMNs infiltration and inflammation, promoting epidermal regeneration and fibroblast proliferation (Cam et al., 2020), Similarly, topically using GQ-11 (a partial/dual PPAR α/γ agonist) and Pioglitazone on the wound of DM mice can also down-regulate IL-1β and TNF-α, promote DW healing (Silva et al., 2019). An in-depth understanding of the relationship between TLR signaling pathways and drug regulation targets, as well as the impact of different types of drugs on drug delivery and efficacy, can provide a feasible direction for the treatment of DW. (Table 3).

**TABLE 3:** Effects of modulating innate immunity pathways in experimental DW.

Innate immune pathway	Knockout mice models	Intervention (agent)	Mechanism
TLR signaling	TLR2^−/−^	—	TLR2/6-MyD88-NF-κb↓
			IL- 1β, TNF-α↓
			DW Healing ↑ Dasu et al. (2010)
	TLR4^−/−^	—	TLR4-NF-κb↓
			IL-6, TNF-α↓
			DW Healing ↑ Dasu and Jialal, (2013)
	Lepr^db/db^	nAChR agonists (nicotine).	TLR2-NF-κb↓
			AMP, IL-6↓
			DW Healing ↑ Kishibe et al. (2018)
	TLR3^−/−^	REG3A	IL-33, REG3A/ RegIIIγ ↑ TLR3–JNK2 ↓ IL-6, TNF-α↓
	JNK2^−/−^	SHP-1 inhibitor (SSG).	DW Healing ↑ Wu et al. (2016)
	Mll1^f/f^Lyz2^Cre+^	TLR4 inhibition	MLL1-mediated H3K4me3 ↑ TLR4-MyD88↓
	TLR4^−/−^	(TAK-242).	DW Healing↑Davis et al. (2020)
NLRP3 inflammasome	Lepr^db/db^ Bitto et al. (2014)	HSWang et al. (2018)	NLRP3 inflammasome-caspase-1-il-1β/il-18 axis↓
		I-κb kinase-β inhibitor (BAY 11–7,082).	IL-1β, IL-18↓
		Purinergic P2X7 receptor inhibitor (brilliant blue G).	DW Healing ↑ Bitto et al. (2014)
	—	PPARα agonists (Fenofibrate).	ROS/TXNIP-NLRP3 inflammasome-caspase-1-il-1β/il-18 axis↓
			DW Healing ↑ Deng et al. (2017)
	—	NLRP3 inhibitor (MCC950).	TLR-4/TLR-9-NF-κb↓
		TLR-4 inhibitor (CLI-095).	ROS/TXNIP-NLRP3 inflammasome-caspase-1-il-1β/il-18 axis↓
		TLR-9 inhibitor (ODN 2088).	IL-1β, IL-18↓
		ROS inhibitor (NAC, dnase I).	DW Healing ↑ Liu et al. (2019)
	Lepr^db/db^	ROS inhibitor (NAC, dnase I).	NLRP3 inflammasome-caspase-1-il-1β/il-18 axis↓ IL-1β, IL-18↓
		IL-1β blocking antibody (IL-1R1).	DW Healing ↑ Mirza et al. (2014)
		Glyburide caspase-1 inhibitor (YVAD).	
cGAS-STING signaling	STING^−/-^	—	Mitochondrial damage-cGAS-STING-IRF3 ↓ ICAM-1↓
			Improve IR, Glucose intolerance Mao et al. (2017)
JAK-STAT signaling	Lepr^db/db^	—	IL-12-Stat4↑
			CCL2-CCR2↑
			DW Healing ↓ Cunnion et al. (2017)
	—	—	IL-6 -il-6rα-jak-stat3↑
			SCOS3, DW Healing ↓ Lee et al. (2019)

Partial abbreviation: TLR: Toll-like receptor, STZ: Streptozotocin, NF-κB: Nuclear factor-κB, IL: Interleukin, TNF-α: Tumor necrosis factor-α, nAChRs: Nicotinic acetylcholine receptors, AMP: Adenosine monophosphate, REG3A: Regenerating islet-derived protein 3A, SHP-1: domain-containing protein-tyrosine phosphatase-1, HS: Heparan sulfate, PPARα: Peroxisome proliferator-activated receptors α, EPC: Endothelial progenitor cells, IGF-1: Insulin-like growth factor-1, TGF-β: Transforming growth factor-β, IR: Insulin resistance.

### Overactivation of NLR Signaling Under Pathological Conditions Promotes Cascading Amplification of the Inflammatory Response in Diabetic wound

NLR is a critical member of the cytoplasmic PRRs family and plays a unique role in the innate immune response. The NLR protein usually exists in an inactivated state in the cytoplasm, which is in an auto-repressed form. When directly or indirectly combine with DAMPs or PAMPs, a conformational change occurs and the NACHT domain is exposed, thereby triggering oligomerization and participating in the activation of multiple signal transduction pathways (Sharif et al., 2019). For example, NOD1 and NOD2 activate NF-κB and MAPK signaling pathways by interacting with RIPK2, while NLRP1, NLRP3, and NLRC4 activate inflammasome through multimerization to produce activated caspase-1 (Platnich and Muruve, 2019). After activation of caspase-1, the inactive pro-IL-1β and pro-IL-18 are cleaved into mature IL-1β and IL-18, thus exerting an immune response and pro-inflammatory effects (Wen et al., 2011). Among them, the NLRP3 inflammasome is a multi-protein complex composed of NLRP3, adaptor protein ASC and pro-caspase-1 (Lamkanfi and Dixit, 2012), which participates in the induction of aseptic inflammation (Vandanmagsar et al., 2011; Wen et al., 2011). Under the pathological conditions of stress and inflammatory, caspase-1 released after the activation of NLRP3 inflammasome can also mediate a rapid programmed cell death pattern characterized by accompanying inflammatory response, called “Pyroptosis” (Wu et al., 2018). Pyroptosis induces the release of a large number of pro-inflammatory factors, eventually forming a cascade of amplified inflammatory responses (Jorgensen and Miao, 2015; Wang S. et al., 2019). In the state of DW, ROS and IL-1β can activate the NLRP3 inflammasome in Mp, promote the maturation and secretion of IL-1β, aggravate local inflammation through positive feedback, and hinder wound healing (Lee et al., 2013; Mirza et al., 2014; Dai et al., 2017). Meanwhile, NETs formed from PMNs which are recruited on the wound can also activate NLRP3 inflammasome through TLR-4/TLR-9/NF-κB and ROS/TXNIP signaling pathways (Liu et al., 2019). Therefore, inhibiting the activities of NLRP3 and caspase-1 and reducing the production of inflammatory factors such as IL-1β, IL-18 may play a role in alleviating inflammation and accelerating DW healing (Bitto et al., 2014). (Figure 3C).

### Therapeutic Targeting of NLR Signaling in Diabetic wound

Currently, researches on NLR mainly focus on NLRP3 inflammasomes and related signaling pathways. Activation of NLRP3 inflammasome requires two steps: Priming and Assembly. Priming mainly targets NF-κB-dependent transcription of NLRP3 and pro-IL-1β(Qiao et al., 2012). Assembly is associated with NLRP3 in mitochondrial relocalization, mitochondrial stress, and cytokines released into the cytoplasm after injury (mtROS, mtDNA, or cardiolipin), ion channel potassium outflow, sodium/calcium influx, and cathepsin release after lysosomal injury (Lamkanfi and Dixit, 2012; Guo et al., 2015). PPARα agonists (such as Fenofibrate) can reduce the expression of TXNIP, NLRP3, and caspase-1 in endothelial precursor cells (EPC) of DM mice and accelerate wound healing of DM mice (Deng et al., 2017). External use of sulfonylureas (such as Glibenclamide) can inhibit the activation of NLRP3, up-regulate IL-10, IGF-1 and TGF-β, down-regulate IL-1β, IL-18, and TNF-α, and as a result down-regulate the pro-inflammatory M1 phenotype, up-regulate the pro-healing M2 phenotype and promote wound healing in DM mice (Mirza et al., 2014). After blocking the P2X7 receptors of the sodium/calcium influx channel, the activities of NLRP3 and caspase-1 are down-regulated, the production of IL-1β and IL-18 is reduced, and the rate of DW angiogenesis and healing is accelerated (Bitto et al., 2014). Blocking the potassium efflux channel (TWIK2) can also down-regulate NLRP3 activation in Mp (Di et al., 2018). Besides, Metformin inhibits NLRP3 activation through the AMPK/mTOR signaling pathway and promotes M2-type polarization of Mp, and is beneficial to Non-DW healing (Qing et al., 2019). Recent studies have found that phosphorylation of NLRP3 inflammasome at the S194 site is an important initiation event for its activation, and blocking NLRP3 phosphorylation by S194A mutation or JNK1 inhibitor can inhibit NLRP3 inflammasome activation (Song et al., 2017). This suggests that preventive regulation of the aggregation of inflammatory cells from upstream may have a beneficial effect on the prevention and treatment of DW. (Table 3).

### Targeting Abnormal Activation of cGAS-STING Signaling Under Metabolic Stress Shows the Potential to Promote Diabetic wound Healing

cGAS-STING signaling was initially thought to activate innate immunity by identifying DNA derived from microorganisms such as viruses or bacteria. However, existing studies have shown that under certain pathological conditions, this signaling pathway can also sense cytoplasmic DNA as a cellular danger signal. Cytoplasmic DNA triggers a STING-dependent inflammatory response and is associated with a variety of severe auto-inflammatory and immune diseases in humans (Ahn et al., 2012), such as Aicardi-Goutières Syndrome (AGS) (Pokatayev et al., 2016), Associated Vasculopathy With Onset in Infancy (SAVI) (Liu et al., 2014), and Systemic Lupus Erythematosus (SLE) (Kato et al., 2018). Besides, abnormal activation of cGAS-STING under mitochondrial dysfunction or metabolic stress can also induce more common diseases, such as obesity (Luo et al., 2018; Bai et al., 2020) and obesity-induced inflammation (Bai et al., 2017), insulin resistance, and glucose intolerance (Mao et al., 2017), Non-Alcoholic Steatohepatitis (NASH) (Yu et al., 2019), Chronic Obstructive Pulmonary disease (COPD) (Qin et al., 2019), Age-related macular degeneration (AMD) (Wu et al., 2019), and Parkinson’s disease (PD) (Sliter et al., 2018), etc. These diseases are often characterized by the excessive signal of interferons and/or cytokines associated with cGAS-STING activation (Li et al., 2013).

Acute pancreatitis (AP).is an acute inflammatory disease characterized by extensive necrosis of pancreatic cells. The DNA released after the death of pancreatic acinar cells activates the STING signal in Mp, promotes the expression of downstream TNF-α and IFN-β lead to aggravating the inflammation and pancreatic injury. DMXAA-induced STING activation can further aggravate the symptoms of AP. At the same time, inhibiting the activity of STING through degrading the released DNA from dead acinar cells by dnaseⅠor inactivate the pathway can both down-regulate the expression of TNF-α and IFN-β, improve the progression of acute inflammation (Zhao et al., 2018). Hidradenitis Suppurativa (HS).is a stubborn and relapsed chronic skin inflammatory disease. Affected by the imbalance of intracellular homeostasis and spontaneous DNA damage caused by replication stress, IFI16 relocates from the nucleus to the cytoplasm. It forms a complex with cGAS, prompting keratinocytes to recognize cytoplasmic DNA to activate STING, and prompting replication stress through cGAS/IFI16-STING positive feedback from chronic and persistent inflammation (Orvain and et al., 2020). It suggests that targeting the cGAS-STING pathway can produce beneficial effects on acute and chronic inflammatory diseases. Adipose tissue-specific knockout of Oxidoreductase-like protein (DsbA-L) can lead to impaired mitochondrial function, promote mtDNA release, and activate the cGAS-STING pathway to cause inflammation and insulin resistance (Bai et al., 2017). Inhibiting STING can down-regulate IL-6, IL-1β, MCP-1, TNF-α, IFNα, and IFNβ, reduce cardiac inflammation and fibrosis in mice with myocardial hypertrophy. Compared with Endoplasmic reticulum stress (ES) inhibitor (4-PBA), ES activator (Tg) pretreatment significantly increases the expression of STING and up-regulates TBK1, IRF3, and NF-κB (Zhang P. et al., 2020). It suggests that ES and mitochondrial damage caused by metabolic stress can also activate the cGAS-STING pathway, and targeting the cGAS-STING pathway has therapeutic potential for the treatment of metabolic diseases (Chung et al., 2019). Finally, knockout STING can improve the systemic insulin resistance and glucose intolerance in high-fat fed mice (Mao et al., 2017), and the analysis of Mp found that STING defect also decreased Mp proinflammatory M1 phenotype (Luo et al., 2018). Finally, Palmitic acid (PA) induces the release of mtDNA into the cytoplasm in a lipotoxic manner, activates the cGAS-STING-IRF3 pathway (Mao et al., 2017), On the one hand, up-regulates the expression of ICAM-1and induces endothelial cell inflammation (Mao et al., 2017), on the other hand, mediates the inactivation of Hippo-YAP pathway of endothelial cells and inhibits angiogenesis (Yuan et al., 2017). This suggests the therapeutic prospect of targeting the cGAS-STING signaling pathway in diabetes and related complications such as DW. (Figure 3D and Table 3).

### Targeted Negative Regulation of JAK-STAT Signaling in Diabetic wound Specific Phases May Promote Healing

JAK-STAT signaling includes four JAKs (JAK1, JAK2, JAK3, and Tyk2).and seven STATs (STAT1, STAT2, STAT3, STAT4, STAT5A, STAT5B, AND STAT6). (Wan et al., 2021), and is essential for the maintenance of homeostasis. Elevated IFN-γ and JAK-STAT1 in obese patients induce adipocyte dysfunction and insulin resistance (McGillicuddy et al., 2009). Knockout STAT4 effectively improved adipose tissue inflammation and insulin resistance in high-fat fed mice (Dobrian et al., 2013), and knockdown of Stat3 activity prevent diabetic glomerulopathy inflammation and abnormal matrix synthesis at an early stage (Lu et al., 2009). A significant increase in expression of STAT4 and the downstream Mp chemokine CCL2 and its receptor CCR2 were also found in early DW, which may be related to the release of IL-12 by pro-inflammatory Mp and the continuous activation of IL-12-STAT4 (Cunnion et al., 2017). It is suggested that STAT4 inhibition can improve the inflammatory response in DW and promote healing.

Cytokines (pro-inflammatory and anti-inflammatory)., growth factors, and chemokines in DW can activate the JAK-STAT pathway, however, they trigger different transcriptional programs. Posttranslational modifications of STAT proteins, such as tyrosine phosphorylation, are essential to ensure differential expression of STAT target genes (Dodington et al., 2018). JAK-STAT signaling is inhibited by SH2-containing phosphatase, protein inhibitors against STATs, and suppressor of cytokine signaling suppressor of cytokine signaling (SOCS). (Wormald and Hilton, 2004; Galic et al., 2014), protein tyrosine phosphatases (PTPs).are also important negative regulators of this signaling pathway (Gurzov et al., 2015). A recent study found that there is nearly 2-fold decreased SOCS3 expression in DW, and greater up to 6-fold IL-6 and IL-6Rα protein expression throughout the majority of the wound healing period, leading to a subsequent increase in phosphorylation of STAT3, this suggests that the apparent upregulation of IL-6 and its receptor in the diabetic skin, as well as inhibition of SOCS3, may lead to increased STAT3 activation and ultimately result in dysregulated inflammation in DW (Lee et al., 2019). Furthermore, STAT3 activation by IL-6 was partly attenuated by Mito-Q, a mitochondrial-targeted antioxidant, suggesting that mtROS potentiates STAT3 signaling in response to IL-6 exposure (Abid et al., 2020), and also suggested that targeted oxidative stress and mitochondrial damage could inhibit the activation of JAK-STAT signals and promote DW healing. IL-6R-mediated gp130 /JAK-STAT3 signal loops are also negatively modulated by SOCS3, SOCS3 is expressed in the epithelium at the edge of the injured wound (Goren et al., 2006; Linke et al., 2010), and SOCS3 overexpressed specifically strongly disturbs DW healing by interfering with keratinocyte proliferation and migration (Linke et al., 2010), However, specific deletion of SOCS3 in keratinocytes delayed healing as well, resulting in the hyperproliferative *epidermis* and prolonged inflammation (Zhu et al., 2008). The research on JAK-STAT regulation immune cells in DW indicated that deregulated immune response in which impaired activation, recruitment and survival of immune cells mediated by downregulation of FOXM1 and STAT3 contribute to the delayed wound healing in DFU (Sawaya et al., 2020). This suggests that we need to further understand the effects of SOCS3 on the positive and/or negative regulation of different effector cells in DW. Furthermore, hyperglycemia up-regulated the expression of PTP1B and inhibited VEGF-induced vascular formation, proliferation, and migration (Zhang et al., 2015), PTP1B activity was significantly elevated in DW, topically applied PTP1B inhibitor may help counterbalance ER stress (Thiebaut et al., 2018) and accelerate DW healing (Zhang et al., 2017; Figueiredo et al., 2020). This may be due to the distinct tissue environments or the competing effects of cytokines that signal through JAK-STAT. IL-6R activates the JAK1/JAK2-STAT3 cascade, IL-10R activates the JAK1/TYK2-STAT3 cascade, also both JAK2 and TYK2 are targets of PTP1B, but PTP1B was not required for inhibition of the proinflammatory receptor IL-6R, PTP1B may be particularly important in regulating IL-10R given that this receptor does not contain a SOCS3-binding site (Pike et al., 2014). In a word, the manipulation of JAK-STAT signaling is a promising therapeutic angle for the treatment of DW. Nonetheless, the pleiotropic biological activities of JAK-STAT signaling imply that targeting therapy to isolated cytokines and/or chemokines at specific stages of the disease might be most beneficial. (Figure 3B and Table 3).

### The Contact Between the Innate Immune Signaling

Just as the factors affecting wound healing are not only single factors, TLR, NLRP3 inflammasome, and cGAS-STING are also associated with DW. First, LPS cannot induce the production of NLRP3 or pro-IL-1β in TLR4 defects or MyD88 and TRIF double-defects cells, and the induced expression of NLRP3 is positively correlated with NF-κB (Bauernfeind et al., 2009). It suggests that the TLR4 pathway and downstream activated NF-κB can regulate the activation of NLRP3, which can regulate the pathologic inflammation associated with NLRP3 inflammasome by targeting TLR. Second, the detection of DNA in the cytoplasm by cGAS-STING triggers lysosomal membrane permeabilization leading to lysosomal cell death (LCD), induces the efflux of potassium, and drives the activation of NLRP3 inflammasome. It suggests that targeting the cGAS-STING-LCD-NLRP3 pathway can improve pathologic inflammation associated with cytoplasmic DNA (Gaidt et al., 2017). Besides, during the activation of typical or atypical inflammasome triggered by inflammatory cell activation, all inflammatory caspases (caspase-1, 4, 5, 11) can cleave cGAS to regulate the cGAS-STING pathway negatively (Wang Y. et al., 2017), and apoptotic caspases (caspase-3, 7) released at the time of apoptosis can also cleave cGAS to prevent unnecessary or excessive immune activation (Ning et al., 2019). It suggests that it is vital to clarify the mechanisms of precise regulation between different innate immune pathways to maintain homeostasis.

Mitochondrial damage or loss of membrane integrity leading to the release or exposure of Mitochondrial components (mtDNA, ROS, cardiolipin) to the cytoplasm, which plays a key regulatory role in the activation of the NLRP3 inflammasome, TLR, cGAS-STING pathway and the formation of NETs. For example, any pathological state that causes mtDNA to leak into the cytoplasm can promote the activation of the cGAS-STING pathway. The appearance of cardiolipin (Iyer et al., 2013; Toksoy et al., 2017), mtDNA, and ROS (Lamkanfi and Dixit, 2012; Guo et al., 2015) in the cytoplasm lead to the activation of the NLRP3 inflammasome. MtDNA activates TLR9 and STING pathways to promote the formation of NETs. These suggest that the pivotal role of mitochondrial homeostasis in innate immune signals, and treatments that promote mitochondrial homeostasis will also accelerate DW healing. (Figure 3).

## Conclusion

Innate immunity is like a double-edged sword. While protecting the body against metabolic stress or injury pressure, there is also a risk of “hurt both enemies and selves”. Therefore, regulating it to maintain a balanced state will play a promoting role in the treatment of DW. Just as the spatial and temporal synchronization in the expression of innate immune cells during the healing process of DW, these different innate immune pathways also have complex connections. Multiple different innate immune pathways may regulate the process of DW, or the transduction of different innate immune pathways may vary at different stages of DW healing. At present, there are few studies based on the crosstalk between multiple signal pathways of the same disease, or the roles of different signal pathways in different periods of the same disease. Therefore, further in-depth study of the differences in the temporal and spatial expression of these innate immune signals will also help us to clarify the pathogenic mechanisms of DW and provide evidence for further prevention and treatment. Besides, activating or inhibiting innate immune signal pathways may improve the disease. However, it may also lead to the proliferation and deterioration of tumor cells or immune deficiency and increased susceptibility. Therefore, whether the activation or inhibition of innate immune pathways has more advantages than disadvantages for DW healing is also worth further discussion.

In this review, the relationship between chronic inflammation and continuously innate immune activation in DW was discussed from the perspective of immune-aging. Firstly, the phenotype changes of innate immune cells under the cell senescent phenotype in DW were identified, such as the feedback loop from delayed activation early and fade delay late of PMNs and oxidative stress aggravate tissue damage, the persistent proinflammatory phenotype of Mp in the metabolic immune microenvironment maintains the release of pro-inflammatory factors. In addition, we discussed several immune-related pathways that trigger inflammation from the perspective of mitochondrial injury and endoplasmic reticulum stress under metabolic stress. Then, make a point that continuous activation of TLR, NLR, and cGAS-STING signals may negatively regulate DW healing. Cytokine and/or chemokine targeting therapy in response to the pleiotropic bioactivity of the JAK-STAT signal. In summary, we provide a series of new potential therapeutic targets for future research on DW.

### Positive Effect of Specialized Pro-Resolving Mediators in Promoting the Timely Resolution of Inflammation and Reconstructing the Self-Limiting Mechanism of Inflammation

As a new therapeutic method in many acute and chronic inflammatory diseases, Specialized pro-resolving mediators (SPMs).has become a research hotspot (Fullerton and Gilroy, 2016). Low levels of circulating SPMs in DM patients give this population the potential to be treated with SPMs (Spite et al., 2014; Brennan et al., 2019). SPMs promote PMNs clearance, inflammation regression, and epithelial tissue repair (Quiros and Nusrat, 2019), local injection of Resolvin D1 (RvD1).in DW can correct the phagocytosis functional defects of Mp, clearance of apoptotic PMNs, and promote wound healing (Tang et al., 2013). Further research has found that RvD1 can also alleviate oxidative stress damage, reduce PMNs infiltration, up-regulate M2 phenotype of Mp by reducing the expression of IL-1β and TNF-α, to promote corneal epithelial wound healing in DM mice (Zhang et al., 2018). Subsequent research also pointed to the role of RvD1 in inhibiting the activation of NLRP3 inflammasome. By inhibiting the activation of NLRP3, ASC, caspase-1, and NF-κB to reduce the production of IL-1β and IL-18, propelling RvD1 to be expected an effective means to alleviate the progression of diabetic retinopathy (Yin et al., 2017). Studies on the other members of the SPMs family such as Resolvin D2 (RvD2). also found similar mechanisms (Lopategi et al., 2019). Maresin-1 (MaR1).promotes the differentiation of Mp to M2 phenotype and accelerates the regeneration of damaged tissue (Abdulnour et al., 2014), besides, MaR1 helps maintain normal mitochondrial function, alleviates the damage to mitochondria from oxidative stress by reducing the production of ROS, and avoids the release of mtDNA (Gu et al., 2018). Recently, the discovery of MAR1-specific binding receptors RORα(Han et al., 2019)and LGR6 (Chiang et al., 2019)also opened a new direction for the research of MAR1 (Im, 2020). A clinical correlation study also found that decreased plasma Mar1 concentration is associated with DFU (Miao et al., 2020), which further suggests that MAR1 may have a therapeutic effect in DW, but the deeper mechanism and the regulation of innate immune pathways need further study.

### Regulating Oxidative Stress and Protecting Mitochondrial Homeostasis

Resveratrol and synthetic sirtuin activators (Maiese, 2015) have a well-established diminished oxidative stress action, may contribute to the conferring effect of reducing chronic low-grade inflammation and promote DW healing, and further studies will develop new therapeutic strategies for DW (Zhang Y. et al., 2020).

### Improve Autophagy Pathway Defects

Endoplasmic reticulum stress-induced autophagy deficiency is associated with chronic inflammation observed in patients with diabetes (Wang et al., 2013), AURKA via Targeting FOXO3a enhanced autophagy of Adipose-Derived Stem Cells (ADSC).promote DW healing (Yin et al., 2020). Exosomes from ADSC can also induce miR-128-3p/SIRT1-mediated autophagy to promote DW healing (Shi et al., 2020). Given the protective role of autophagy in the inhibition of apoptosis and the overactivation of NLPR3 and cGAS-STING, targeted autophagy will also become an effective means in DW healing.

### Suppression of Ferroptosis

Targeting Ferroptosis in immune cells and immunotherapy has shown significant effects (Wang W. et al., 2019). Recent studies have shown that iron overload can induce Ferroptosis in Mp (Wang Q. et al., 2017), GPX4 is a key protein in inhibiting Ferroptosis, and RSL3, as an inhibitor of GPX4, can induce Ferroptosis in M2 Mp (Kapralov et al., 2020). Some Ferroptosis-related metal transporters such as ZIP14 (SLC39A14).may also play a regulatory role in the immune system (Yu et al., 2020). Therefore, elucidating the molecular mechanism of Ferroptosis in immune cells will contribute to the development of new therapeutic strategies for DW, which is a promising research direction.

In a word, it is foreseeable that with the strategy of “promoting the timely regression of inflammation and reconstructing the self-limiting mechanism of inflammation”, by reprogramming immune cells rather than inhibiting the inflammatory response, finding the spatial and temporal synchronization in innate immune response and inflammatory response during the process of DW healing, will be a promising area of research with the potential to identify novel therapeutic strategies.
